# Discriminative Ability and Associations of Sarcopenia Using Point-of-Care Ultrasound with Functional, Mobility and Frailty Status in Older Inpatients

**DOI:** 10.3390/jcm14051603

**Published:** 2025-02-27

**Authors:** Rahel Zehnder, Martin Schimmel, Lisa Meyer, Miriam Kömeda, Andreas Limacher, Anna K. Eggimann

**Affiliations:** 1 Medical Faculty, University of Bern, 3012 Bern, Switzerland; 2 Department of Reconstructive Dentistry and Gerodontology, School of Dental Medicine, University of Bern, 3010 Bern, Switzerland; martin.schimmel@unibe.ch; 3 Department of Orthopaedic Surgery and Traumatology, Inselspital, Bern University Hospital, University of Bern, 3010 Bern, Switzerland; 4 Department of Geriatrics, Inselspital, Bern University Hospital, University of Bern, 3010 Bern, Switzerland; 5 Department of Clinical Research, University of Bern, 3010 Bern, Switzerland; 6 Swiss Paraplegic Research, 6207 Nottwil, Switzerland

**Keywords:** POCUS, cross-sectional area, thickness, EWGSOP2, predictive validity, muscle mass, geriatric assessment

## Abstract

**Background/Objectives:** We aimed to assess the discriminative ability of point-of-care ultrasound (POCUS) of the rectus femoris (RF) to detect sarcopenia and to examine associations of these sarcopenia measures with functional, mobility, and frailty status among older inpatients. **Methods:** Data were analysed from 161 patients aged 70 years and older consecutively admitted to a tertiary geriatric rehabilitation hospital between October and December 2023. The RF thickness and cross-sectional area (CSA) were measured using POCUS applying validated cut-offs. Ability of muscle ultrasound to detect sarcopenia based on bioelectrical impedance analysis (BIA) as the reference standard was calculated using receiver operating characteristics analyses (ROC). Second, associations of sarcopenia measures based on either the ultrasonographic RF thickness, or the RF cross-sectional area with functional, frailty, and mobility status were analysed using multivariable logistic regression analyses. **Results:** Mean age was 84.0 years (standard deviation (SD) 6.1 years) and 64.4% were women. Overall, 31 (19.3%) patients had sarcopenia based on low grip strength and low muscle mass using the BIA. The mean ultrasonographic RF thickness and CSA were 13 mm (SD 4.1) and 4.3 cm^2^ (SD 1.7), respectively. Correlation coefficients of the RF thickness with BIA-muscle mass were r = 0.52 in males, versus r = 0.40 in females. Both sarcopenia measures using the RF thickness and CSA were positively associated with functional (adjusted odds ratio (OR) 9.3 (95% CI 3.7–23.4) and 9.2 (3.6–23.7)) and frailty status (OR 4.0 (95% CI 2.1–12.1) and 4.3 (1.8–10.4)). None of the sarcopenia measures were significantly associated with mobility status. **Conclusions:** Rectus femoris thickness and CSA measured by POCUS showed a fair discriminative ability to detect sarcopenia based on BIA, suggesting that BIA and POCUS measure different aspects of muscle health. A strong association between sarcopenia based on POCUS and functional and frailty status suggest the potential utility of POCUS in the diagnostic evaluation of sarcopenia among older hospitalised patients; however, further study is required. Research should focus on establishing valid sex-specific cut-offs for grip strength and muscle mass, with the ultimate goal of developing a low-cost, bedside, and sensitive diagnostic toolkit for detecting sarcopenia in older patients.

## 1. Introduction

Sarcopenia is a highly prevalent clinical condition with a significant disease burden, along with substantial social and economic impacts [1,2,3]. According to Dent et al. (2018), 6–20% of adults over the age of 65 are affected by sarcopenia [4]. The prevalence is expected to increase as the proportion of older adults in the population grows [5]. Clinically, sarcopenia elevates the risk of falls and fractures [6,7], and leads to functional decline [8,9].

A recent Delphi consensus from the Global Leadership Initiative in Sarcopenia (GLIS) states that muscle mass is accepted as a key component of sarcopenia [10]. Therefore, it is crucial to have a valid and feasible method to assess muscle mass in older patients. Currently, muscle mass is assessed based on the European Working Group on Sarcopenia in Older People (EWGSOP2) [5] using different methods, including bioelectrical impedance analysis (BIA), dual x-ray absorptiometry (DXA), computed tomography (CT), and magnetic resonance imaging (MRI). However, these methods have limitations, including issues related to radiation, accessibility, contraindication (e.g., pacemaker BIA altered by costs), and validity (altered by fluid status in BIA) [5].

Similarly, availability of DXA is limited in most countries. Switzerland, a high-income country with an advanced health care system even has limited machines [11]. Moreover, DXA machines equipped with full-body scan (for calculation of muscle mass) are larger in size and not available as a mobile unit [12].

As a result, there has been an effort to simplify sarcopenia diagnosis by utilizing point-of-care ultrasound (POCUS). Compared to other methods, POCUS has several merits compared to conventional methods, such as highly accessible, bedside access, low-cost, no contraindication, no radiation, and user-friendly. Notably, systematic reviews by Nijholt et al. [10] and Fu et al. [13] showing that ultrasound is a valid and reliable method to assess muscle size, particularly of the lower limb, such as the rectus femoris (RF) [13].

However, there are limited studies investigating the role of POCUS for diagnosis of sarcopenia in geriatric patients. A recent scoping review by our study group concluded that data on ultrasound to identify sarcopenia is particularly lacking in hospitalised older patients [14]. This review found promising data of POCUS in particular for the RF thickness and cross-sectional-area in geriatric patients. Therefore, we aimed to fill this gap in knowledge by evaluating the discriminative ability of the RF ultrasound to identify sarcopenia based on BIA and to assess associations of these measures regarding functional, mobility, and frailty status in older inpatients in a post-acute care setting.

## 2. Materials and Methods

### 2.1. Study Design

The study was conducted from 2 October to 30 November 2023 consecutively including all patients admitted to the Geriatric Rehabilitation Hospital in Belp, Switzerland. The hospital admission criteria were as follows: age ≥ 70 years, transfer directly from acute care to the rehabilitation centre, and potential for functional improvement before admission during their stay at the rehabilitation centre. Patients who were isolated due to infectious diseases were not included.

The study was approved by the Ethics Committee in Bern, Switzerland (Req-2023-00665). Following research regulations about human subjects’ health-related data, we analysed anonymised data so individuals could not be identified.

A multidimensional geriatric assessment was performed as a clinical routine by trained assessors upon admission. The assessment included a mini mental status examination (MMSE) (cognitive deficit ≤ 26) [15] and a clock test (deficit in executive and visuospatial function ≤ 5) [16], a 5-item geriatric depressionscale (GDS-5) (depressive symptoms ≥ 2 points) [17], 4 m gait speed test (low gait speed ≤ 0.8 m/s) [18], Nutritional Risk Screening 2002 (NRS; nutritional deficit ≥ 3 points) [19], a Jäger visual acuity table for near vision (vision impairment < 20/70 for both eyes) [20], and a whisper test for hearing (hearing impairment < 6 for both sides combined) [21]. Moreover, the geriatric assessment included other assessments described in detail in Section 2.2.

### 2.2. Sarcopenia Assessment

Sarcopenia was defined according to the European Working Group on Sarcopenia (EWGSOP2) consensus based on low muscle strength and low muscle mass [5].

In summary, the following three sarcopenia measures were investigated: (1) sarcopenia based on low grip strength and low muscle mass using BIA, (2) sarcopenia based on low grip strength and low muscle mass using POCUS to measure the RF thickness, and (3) sarcopenia based on low grip strength and low muscle mass using POCUS to measure the RF CSA. Details of these assessments are provided in the following paragraphs.

Muscle strength was evaluated by the grip strength using the Martin Vigorimeter for the dominant hand in three trials with a 30-s break between each trial. The best of the three trials was used and converted from kPa to kg using the Neumann conversion table. Cut-off values from the EWGSOP2 were applied to define low grip strength (<16 kg for women and <27 kg for men) [5,22].

Muscle mass was measured by both BIA and POCUS in all patients by a trained assessor (RZ). A multisegmental BIA device (BIACORPUS RX 4004M, MEDI CAL HealthCare GmbH) was used to measure muscle mass [23]. Details of the BIA measurement are described by Stuck et al. (2022) [23]. An integrated software (Bodycomposition V9.0, MEDI CAL HealthCare GmbH, Bonn, Germany) calculated the appendicular skeletal muscle index (ASMI) (kg/m^2^)21 based on the Sergi equation [24]. All patients having a contraindication (e.g., a pacemaker or internal cardioverter defibrillator) were excluded from the BIA. Cut-off values for low muscle mass were based on the EWGSOP2 guidelines (<5.5 kg/m^2^ for women and <7 kg/m^2^ for men) [5,25].

A point-of-care ultrasound was performed to measure the RF thickness and cross-sectional area (CSA) using SonoSite iViz, Fujifilm, and a linear array transducer (SonoSite iViz L38, Fujifilm, Tokyo, Japan) using a linear transducer (10–5 MHz linear). The patient was in a supine position with an extended and relaxed knee position. The RF thickness and CSA were measured in the arithmetic middle between the greater trochanter and the upper edge of the patella of the right leg using a tape measure [26]. If the right extremity was amputated or non-accessible due to a wound, the left leg was measured instead. The transducer was placed at the marked position using the slightest pressure. The ventrodorsal thickness of the rectus femoris muscle was measured, and an integrated ellipse program was used to determine the cross-sectional area (CSA) of the rectus femoris. For the second measurement of the RF thickness and CSA the transducer was repositioned at the same position. The mean for both values was taken for analysis [26,27]. As a cut-off for low RF thickness and RF CSA and consequently low muscle mass, values of a previous study were taken. In their survey, Ozturk et al. (2022) defined outpatients with low RF thickness as values ≤ 13 mm for women and ≤15.5 mm for men, as well as low RF CSA as values ≤ 4.3 cm^2^ for women and ≤5.2 cm^2^ for men [26].

### 2.3. Clinical Outcomes

Mobility status was measured using the de Morton Mobility Index (DEMMI) [28] upon admission. The cut-off of the DEMMI defining impaired mobility status was set at ≤40 points according to a previous study [29].

Functional status was evaluated by the Extended Barthel Index upon admission. The cut-off for an impaired functional status was set at the lowest quartile (≤31.5 points) [30].

Frailty was assessed with the German version of the Clinical Frailty Scale (CFS) upon admission classifying CFS scores of 1–4 as non-frail, and 5–8 as frail [31,32].

### 2.4. Statistical Analyses

Characteristics of the study population are presented by absolute and relative frequencies or by mean with standard deviation for categorical and continuous variables, respectively. Correlations of the ultrasound for RF thickness and RF CSA with muscle mass based on a BIA was assessed using the Pearson correlation coefficient. The ability of the ultrasound parameters to detect sarcopenia based on low grip strength and low muscle mass using a BIA (EWGSOP2) as the reference standard was calculated using receiver operating characteristics curve (ROC) analyses with a 95% confidence interval (95% CI), sensitivity, specificity, and positive and negative predictive values (PPV, NPV). Associations of sarcopenia measures based on ultrasound and bioelectrical impedance analyses(BIA) were calculated for functional status, frailty, and impaired mobility using univariable and multivariable logistics analyses. In a sensitivity analysis, we used different cut-offs for low RF thickness and low RF CSA using the Youden index to calculate the optimal cut-off being associated with sarcopenia based on the BIA. The resulting Youden index derived cut-offs for RF thickness were ≤13.7 mm for males and ≤11.5 mm for females, and for RF CSA were ≤4.4 cm^2^ for males and ≤3.1 cm^2^ for females. Based on these Youden derived sarcopenia definitions we calculated univariable and multivariable logistic regression models for the three outcomes. 

Measures of repeatability for ultrasound of the RF thickness and CSA (intra-class correlation (ICC)) were calculated using one-way analysis-of-variance (ANOVA) models. Bland–Altman plots were generated displaying the differences between measurements 1 and 2 of the ultrasonographic rectus femoris against the mean of the two rectus femoris measurements. All analyses were computed using Stata Version 16.1 (StataCorp LLC, College Station, TX, USA). A *p*-value of <0.05 was considered statistically significant.

## 3. Results

The 161 patients included in this study had a mean age of 84.0 years (SD 6.1), and 64.6% were women (Table 1). Overall, 65 (40.4%) of the patients were frail according to the CFS, and 53 (32.9%) had malnutrition based on the NRS. Regarding muscle health, 29.2% had low grip strength, and 43.1% had low muscle mass using the BIA, resulting in a total of 31 patients (19.3%) suffering from sarcopenia based on a BIA and low grip strength. Of the 161 individuals, 10 patients did not have a valid BIA measurement due to contraindication.

Mean ultrasonographic RF thickness and CSA (n = 161) were 13.0 mm (SD 4.1) and 4.3 cm^2^ (SD 1.7), respectively. The left leg was measured in 2/161 patients, as the right leg was not accessible. Overall, 32 patients (19.9%) were classified as sarcopenic based on low RF thickness and low grip strength, and 30 (18.6%) based on low RF CSA and low grip strength, respectively.

**Figure 1:**
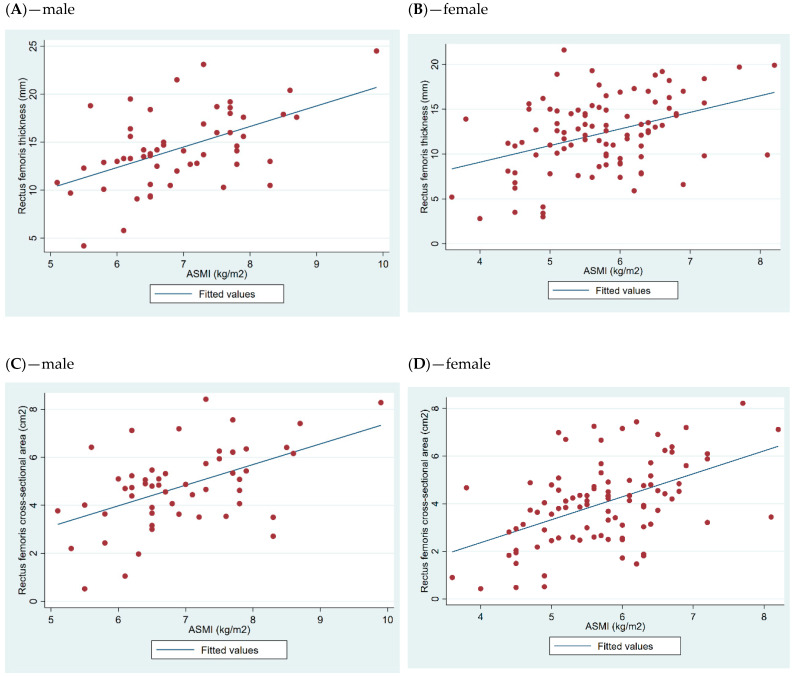
Scatter plot and fitted line for ultrasound rectus femoris and ASMI measured by BIA stratified by sex (n = 151).

For males, the RF thickness had a correlation coefficient of r = 0.52 with muscle mass based on BIA, whereas females had a correlation coefficient of r = 0.40. Similarly, the correlation coefficient of RF CSA was r = 0.50 for both males and females (Figure 1).

The RF thickness and RF CSA stratified by sarcopenia status based on the BIA for women and men are shown in the supplementary information, displaying the difference between sarcopenic and non-sarcopenic individuals. In male patients, the mean RF thickness significantly differed between sarcopenic and non-sarcopenic individuals (12.0 mm; 95% CI, 9.9–14.0 in sarcopenia vs. 15.6 mm; 95% CI, 14.4–16.7 in non-sarcopenia, *p* < 0.01). In female patients, there was no statistically significant difference in the mean RF thickness between sarcopenic and non-sarcopenic participants (11.2 mm; 95% CI, 8.0–14.3 for sarcopenia vs. 12.4 mm; 95% CI, 11.6–13.3 for no sarcopenia, *p* = 0.3)

The predictive ability of the ultrasonographic rectus femoris parameters to detect sarcopenia based on low grip strength and BIA as a reference standard are displayed in Table 2. ROC analyses showed an area under the curve of 0.64 (95% CI 0.56–0.72) for the ultrasonographic RF thickness and 0.61 (95% CI 0.53–0.70) for the RF CSA to detect sarcopenia based on low grip strength and BIA.

Table 3 shows the associations of sarcopenia measures with impaired functional status, frailty, and impaired mobility status at admission. Thereby, sensitivity, specificity, positive predictive value, negative predictive value, area under the curve, and odds ratios are listed for the three sarcopenia measures. All three sarcopenia measures based on BIA, RF thickness, and CSA were positively associated with functional status, but sarcopenia measures based on the ultrasound RF thickness and CSA showed a stronger association (adjusted odds ratio (OR) 9.3; 95% CI, 3.7–23.4 and 9.2; 95% CI, 3.6–23.7, respectively). Similarly, RF thickness and CSA were significantly associated with frailty status (OR 4.0; 95% CI, 2.1–12.1 and 4.3; 95% CI, 1.8–10.4, respectively). None of the sarcopenia measures was significantly associated with mobility status. Sensitivity analyses for the associations of sarcopenia applying the Youden cut-off for the RF thickness and CSA are shown in the supplementary information.

Concerning repeatability of the ultrasonographic measurements (intra-rater), mean difference of the RF thickness measurements 1 and 2 was 0.05 (SD 0.92) mm. Mean difference of the RF CSA measurements 1 and 2 was −0.07 (SD 0.44) cm^2^. Intraclass correlation coefficient (ICC) for RF thickness was 0.98 (95% CI 0.97–0.98) and RF CSA 0.96 (95% CI 0.95–0.97). Bland–Altman plots of RF thickness and CSA, respectively, are displayed in the supplementary information.

## 4. Discussion

Overall, we identified a substantial proportion of patients with sarcopenia based on low muscle mass BIA and low grip strength. The discriminative ability of the ultrasonographic RF parameters for detecting sarcopenia based on BIA showed areas under the curve of 0.64 for RF thickness and 0.61 for RF CSA. Both the RF thickness and CSA were positively associated with functional status and frailty but not with mobility status.

Overall, the prevalence of sarcopenia we found in our study (19.3%) falls within the range reported by other European studies, which show varying prevalences from 13.4% to 38.7% [24,27]. This wide range may be attributed to differences in the study populations, as noted by Dent et al. [4] For example, Ozturk et al. examined geriatric outpatients [26] while Rustani et al. focused on convalescent inpatients [27]. Additionally, Rustani et al. used anthropometric measures, such as mid-arm muscle circumference, which involves measuring mid-arm circumference and tricipital skinfold, instead of BIA to assess muscle mass in sarcopenia [32]. This method might have led to an underestimation of muscle mass and, consequently, an overestimation of sarcopenia prevalence due to the variability of skin elasticity with age and sex, as summarised by Nösslinger et al. [34].

Concerning RF thickness, we observed lower values of 13.0 mm (SD 4.1) than a previous study by Berger et al. reporting a mean RF thickness of 18.2 mm (SD 2.3) for women and 21.6 mm (SD 3.1) for men [35]. This difference could be attributed to the varying study populations, such as geriatric inpatients versus older community-dwelling adults. Ozturk et al., whose cutoff values we used, also reported higher values, with RF thickness averaging 12.2 mm (SD 2.6) for sarcopenic individuals and 14.6 mm (SD 2.9) for non-sarcopenic individuals [26].

We further found a fair to moderate diagnostic accuracy when comparing sarcopenia based on ultrasound versus BIA. The ROC values in our study are in line with the findings of Ozturk et al. [26]. One possible explanation for these low ROC values is that POCUS of the rectus femoris may measure something different about the muscle as an organ compared to muscle mass measured by BIA. This raises the broader question of whether POCUS should be compared to BIA. Looking back in history, ultrasound faced a similar challenge when it was compared to the gold standard of X-ray. Over time, it became clear that, while ultrasound has its own advantages and disadvantages, it should not be directly compared to X-ray. Instead, the focus should be on its ability to predict diseases or clinical outcomes.

Another finding of our study was the association between sarcopenia based on ultrasound and both functional and frailty status. By contrast, we did not find an association between sarcopenia and mobility status. Trends and the wide confidence interval for associations with mobility status suggest that the lack of a detected effect may be due to insufficient power (type II error). Overall, the study extends the findings of previous studies by Rustani et al. [27] and Ozturk et al. [26], who only established an association between ultrasound RF measurements and sarcopenia without considering the practical implications for patients in their everyday lives, as reflected by their functional and frailty status. At the same time, this research is congruent with the data of Hafizoğlu et al. showing an association between CFS and RF thickness and RF CSA in older adults with type 2 diabetes mellitus [36], underlining the data from Yau et al. who investigated perioperative RF muscle ultrasound and frailty scores [37]. These findings partly support the investigation of Mijnarends et al., who stated that there are no significant correlations between muscle mass and the activities of daily living (ADL) but significant correlations with muscle strength and physical performance parameters [1].

Our study has some limitations. First, this is a single-site study with a limited study population of geriatric patients in post-acute care. Therefore, results cannot be directly transferred to other settings or countries limiting generalisability. Second, we based the sarcopenia definition on the EWGSOP2 guidelines and applied cut-offs by Ozturk et al. [26] for ultrasound. Thus, our results may not apply to other sarcopenia definitions and/or cut-off definitions. In a sensitivity analysis, we applied other cut-offs than Ozturk for muscle mass based on POCUS. Further study needs to determine most valid cut-offs for muscle mass and grip strength. Third, we performed subgroup analyses stratified by sex, but did not perform further subgroup analyses to limit type I error. Due to lacking power, we cannot make conclusive statements on subgroups. Fourth, POCUS was performed by a single clinical assessor, allowing for the analysis of intra-rater reliability, which demonstrated a reliable ICC. However, we cannot make any conclusions about inter-rater reliability. Finally, we standardised POCUS to a strict protocol defining side, location, and other details of measurement. Therefore, results are applicable to similar protocols of POCUS, but not to less detailed or different standards.

From a clinical perspective, establishing a universally accessible assessment method for sarcopenia with standardised cut-off values would be highly beneficial. Our study suggests that POCUS can assess a larger number of patients to screen for sarcopenia compared to traditional methods like BIA, as ultrasound is free from contraindications. Specifically, BIA could not be performed in 10/161 (6.2%) patients based on contraindications to the BIA, whereas POCUS could be performed in all patients. On the other hand, CT/MRI/DXA machines are rarely available in geriatric rehabilitation settings, thus BIA provides an accessible, easy, and cost-effective alternative. Additionally, our study found correlations between functional status and frailty, both of which are crucial factors in the discharge management of older patients.

It is noteworthy that a higher percentage of patients were identified with low muscle mass using ultrasound compared to BIA. However, the prevalence of sarcopenia at the population level remained similar. This indicates that, despite low muscle mass, grip strength measurements based on the EWGSOP2 criteria did not lead to a sarcopenia diagnosis. Specifically, our subgroup analyses by sex suggest that the choice of cut-offs to define low grip strength needs special attention in female patients, as we found lower than estimated prevalence of low grip strength in women applying the ESWSGSOP2 cut-offs. These findings highlight the need for a consensus on the most appropriate cut-off values not only for muscle mass but for muscle strength. The lower-than-expected prevalence of sarcopenia in female patients specifically indicates that cut-offs for both muscle mass and grip strength must be carefully revised for female patients.

Finally, further research is needed to explore the trajectories of muscle mass using POCUS. Measurements taken during intensive therapy sessions could potentially serve as a motivational tool for patients, as they would be able to quantitatively track and visualise their muscle mass through follow-up assessments, encouraging continued engagement in exercise.

Our findings suggest that both the ultrasonographic RF thickness and the CSA have moderate discriminative ability for detecting sarcopenia using BIA. Of note, both the RF thickness and the CSA were significantly associated with functional and frailty status, underscoring the potential of POCUS in the diagnostic work-up of sarcopenia in older patients. Establishing accurate cut-off values for grip strength and muscle mass using POCUS is essential to developing a practical, portable, and cost-effective sarcopenia detection toolkit, enabling targeted interventions for older patients. Special attention should be given to female patients to ensure that the selected cut-off values create a highly sensitive screening tool.

## Figures and Tables

**Table 1:** Clinical characteristics of patients (n = 161).

		Overall (n = 161)	Men (n = 57)	Women (n = 104)
Demographics				
	Age, years, mean (SD)	84.0 (6.1)	83.6 (6.6)	84.2 (5.8)
	Weight, kg, mean (SD)	68.5 (15.9)	77.5 (14.4)	63.5 (14.4)
	Height, cm, mean, (SD)	165.5 (15.8)	173.8 (6.4)	160.8 (17.1)
	BMI, kg/m^2^, mean (SD)	24.9 (5.2)	25.6 (4.2)	24.5 (5.6)
Geriatric assessment				
	CIRS, median (IQR)	19 (16–22)	19 (16–23)	19 (16–21.25)
	EBI, median (IQR)	38 (30–45)	37 (28–46)	39 (32–45)
	CFS, median (IQR)	4 (3–5)	4 (3–5)	4 (4–5)
	Frail ^(a)^, n (%)	65 (40.4)	25 (43.9)	40 (38.5)
	MMSE, median (IQR)	25 (21–27)	24 (20–27)	25 (22–27)
	Clock test, median (IQR)	4 (2–6)	4 (2–7)	3.5 (2–5)
	Cognitive impairment ^(b)^, n (%)	95 (59.0)	36 (63.2)	59 (56.7)
	GDS-5, median (IQR)	1 (0–1)	1 (0–1)	1 (0–2)
	Vision impairment ^(c)^, n (%)	14 (8.7)	7 (12.3)	7 (6.7)
	Hearing impairment ^(d)^, n (%)	104 (64.6)	40 (70.2)	64 (61.5)
	NRS, median (IQR)	3 (3–4)	3 (2–4)	3 (3–4)
	Malnutrition ^(e)^, n (%)	53 (32.9)	19 (33.3)	34 (32.7)
	Gait speed, m/s, mean, (SD)	0.6 (0.3)	0.6 (0.3)	0.6 (0.3)
	Low gait speed ^(f)^, n (%)	110 (68.3)	39 (68.4)	71 (68.3)
	Grip strength, kg, mean (SD)	24.2 (10.2)	30.5 (11.7)	20.8 (7.2)
	Low grip strength ^(g)^, n (%)	47 (29.2)	21 (36.8)	26 (25.0)
	ASMI, kg/m^2^, mean (SD) ^m)^	6.2 (1.8)	6.9 (1.8)	5.7 (1.7)
	Low ASMI ^(h)^, n (%)	66 (43.1)	30 (52.6)	36 (34.6)
	Sarcopenia based on BIA low muscle mass ^(i)^, n (%) ^(m)^	31 (19.3)	18 (34.0)	13 (13.3)
Ultrasound parameters				
	RF thickness, mm, mean (SD)	13.0 (4.1)	14.2 (4.1)	12.4 (4.1)
	Low RF thickness ^(k)^, n (%)	92 (57.1)	36 (63.2)	56 (53.9)
	Sarcopenia based on low RF thickness, n (%) ^(n)^	32 (19.9)	17 (29.8)	15 (14.4)
	RF cross-sectional area, cm^2^, mean (SD)	4.3 (1.7)	4.8 (1.7)	4.0 (1.6)
	Low RF cross-sectional area ^(l)^, n (%)	94 (58.4)	36 (63.2)	58 (55.8)
	Sarcopenia based on low RF CSA, n (%) ^(o)^	30 (18.6)	16 (28.1)	14 (13.5)

Abbreviations: SD, standard deviation; n, number; BMI, body mass index; CIRS, cumulative illness rating scale; IQR, interquartile range; EBI, Extended Barthel Index; CFS, clinical frailty scale; MMSE, mini mental status examination; GDS-5, 5-item geriatric depression scale; NRS, nutritional risk screening; ASMI, appendicular skeletal muscle mass index; RF, rectus femoris; CSA, cross-sectional area. ^(a)^ Frail ≥ 5 of the CFS [31,32]. ^(b)^ Cognitive impairment ≤ 26 points in the MMSE [15]. ^(c)^ Vision impairment: Jäger visual acuity table < 20/70 for both eyes [20]. ^(d)^ Hearing impairment: whisper test correct answers < 6 out of 6 both ears combined [21]. ^(e)^ Malnutrition defined as ≥3 points NRS 2002 [19]. ^(f)^ Low gait speed defined as ≤0.8 m/s [5,33]. ^(g)^ Low grip strength using the Martin Vigorimeter < 16 kg for women, <27 kg for men [5,22]. ^(h)^ Low ASMI using BIA < 5.5 kg/m^2^ for women and <7 kg/m^2^ for men [5,25]. ^(i)^ Sarcopenia defined as low grip strength ^(g)^ and low ASM using BIAI ^(h)^. ^(k)^ Low RF thickness ≤ 13.0 mm for women and ≤15.5 mm for men [26]. ^(l)^ Low RF CSA ≤ 4.3 cm^2^ for women and ≤5.2 cm^2^ for men [26]. ^(m)^ n = 151, 10 individuals missing due to contraindications for BIA. ^(n)^ Sarcopenia with RF thickness: low grip strength and low RF thickness. ^(o)^ Sarcopenia with RF thickness: low grip strength and low RF CSA.

**Table 2:** Discriminative ability of ultrasonographic rectus femoris parameters to detect sarcopenia based on BIA (EWGSOP2) (n = 151).

	Cut-Off Definitions ^(a)^	Sensitivity	Specificity	PPV	NPV	AUC (95% CI) ^(b)^
RF thickness	Male ≤ 15.5 mm Female ≤ 13.0 mm	25/31 (80.7%)	57/120 (47.5%)	25/88 (28.4%)	57/63 (90.5%)	0.64 (0.56–0.72)
RF cross-sectional area	Male ≤ 5.2 cm^2^ Female ≤ 4.3 cm^2^	24/31 (77.4%)	55/120 (45.8%)	24/89 (27.0%)	55/62 (88.7%)	0.61 (0.53–0.70)

Abbreviations: BIA, bioelectrical impedance analysis; EWGSOP2, European working group on sarcopenia in older people; RF, rectus femoris; PPV, positive predictive value; NPV, negative predictive value; AUC, area under the curve; CI, confidence interval. Number of individuals = 151 (see methods, discussion). ^(a)^ Cut-off definitions for ultrasonographic rectus femoris parameters based on Eggimann et al. (2024) and Ozturk et al. (2022) [14,26]. ^(b)^ AUC was calculated applying the indicated cut-off definitions of RF thickness and RF CSA, respectively.

**Table 3:** Associations of sarcopenia measures with impaired functional status, frailty, and impaired mobility status upon admission (n = 161).

	Unadjusted OR (95% CI)	Adjusted OR (95% CI) ^(e)^	AUC (95% CI)	Sensitivity n/n%		Specificity n/n%		PPV n/n	%	NPV n/n	%
Sarcopenia based on ultrasound rectus femoris thickness											
Impaired functional status ^(a)^	9.2 (3.8–22.1)	9.3 (3.7–23.4)	0.73 (0.64–0.83)	19/30	63.3%	106/126	84.1%	19/39	48.7%	106/117	90.6%
Frailty ^(b)^	5.3 (2.3–12.4)	4.0 (2.1–12.1)	0.70 (0.61–0.79)	23/32	71.9%	87/129	67.4%	23/65	35.4%	87/96	90.6%
Impaired mobility status ^(c)^	2.2 (0.9–5.1)	2.4 (1.0–5.8)	0.59 (0.49–0.70)	13/28	48.2%	84/120	70.0%	13/49	26.5%	84/90	85.7%
Sarcopenia based on ultrasound rectus femoris cross-sectional area											
Impaired functional status ^(a)^	9.2 (3.7–22.6)	9.2 (3.6–23.7)	0.73 (0.64–0.84)	18/28	64.3%	107/128	83.6%	18/39	46.2%	107/117	91.5%
Frailty ^(b)^	4.6 (2.0–10.9)	4.3 (1.8–10.4)	0.68 (0.59–0.77)	21/30	70.0%	87/131	66.4%	21/65	32.3%	87/96	90.6%
Impaired mobility status ^(c)^	2.1 (0.9–5.1)	2.4 (0.9–5.8)	0.59 (0.50–0.70)	12/25	48.0%	85/122	69.7%	12/49	24.5%	85/98	86.7%
Sarcopenia based on BIA ASMI ^(d)^											
Impaired functional status ^(a)^	5.2 (2.2–12.4)	5.3 (2.1–13.6)	0.67 (0.57–0.77)	15/29	51.7%	97/117	82.9%	15/35	42.9%	97/111	87.4%
Frailty ^(b)^	5.1 (2.1–12.1)	5.2 (2.1–13.1)	0.69 (0.60–0.78)	22/31	71.0%	81/120	67.5%	22/61	36.1%	81/90	90.0%
Impaired mobility status ^(c)^	1.5 (0.6–3.6)	1.7 (0.7–4.6)	0.55 (0.43–0.65)	10/25	40.0%	78/113	69.0%	10/45	22.2%	78/93	83.9%

Abbreviations: n, number; OR, odds ratio; CI, confidence interval; AUC, area under the curve; PPV, positive predictive value; NPV, negative predictive value; DEMMI, de Morton mobility index; BIA, bioelectrical impedance analysis; ASMI, appendicular skeletal muscle mass index. Number of individuals = 151 (see methods, discussion). ^(a)^ Impaired functional status defined as extended Barthel index ≤31.5 points upon admission. n = 5 missing. ^(b)^ Frailty defined as Clinical Frailty Scale ≥5 points; ^(c)^ Impaired mobility status defined as DEMMI ≤40 points upon admission. n = 14 missing. ^(d)^ n = 151 (n = 10 missing, BIA could not be performed due to contraindications). ^(e)^ Adjusted for age (continuous) and sex (female vs. male).

## Data Availability

All data are provided in the manuscript and supplementary information.

## Supplementary Materials

The following supporting information can be downloaded at: https://www.mdpi.com/article/10.3390/jcm14051603/s1, Figure S1: Ultrasonographic rectus femoris parameters stratified by sarcopenia status and by sex (n = 151) (n = 10 missing, BIA could not be performed due to contraindications). (A) rectus femoris thickness, (B) rectus femoris Cross-sectional area; Figure S2: Bland Altman Plots of repeatability of measurements of POCUS (intra-rater). (A) Rectus femoris thickness; (B) Rectus femoris Cross-sectional area (CSA); Table S1: Sensitivity analyses. Association of sarcopenia measures applying Youden cut-offs ^(e)^ with impaired functional status, frailty, and impaired mobility status upon admission (n = 161).

## Abbreviations

The following abbreviations are used in this manuscript:

ADL	Activities of daily living
ASMI	Appendicular skeletal muscle mass index
AUC	Area under the curve
BIA	Bioelectrical impedance analysis
BMI	Body mass index
CFS	Clinical Frailty Scale
CI	Confidence interval
CIRS	Cumulative illness rating scale
CSA	Cross-sectional area
CT	Computer tomography
DEMMI	de Morton Mobility Index
EBI	Extended Barthel Index
EWGSOP2	European working group on sarcopenia in older people
GDS-5	5-item geriatric depression scale
GLIS	Delphi Consensus from the Global Leadership Initiative in Sarcopenia
IQR	Interquartile range
MRI	Magnet resonance imaging
NPV	Negative predictive value
NRS	Nutritional risk screening
OR	Odds ratio
POCUS	Point-of-care ultrasound
PPV	Positive predictive value
RF	Rectus femoris muscle
ROC	Receiver operating characteristic curve analysis
SD	Standard deviation
