# Supplementary material for: Discriminative Ability and Associations of Sarcopenia Using Point-of-Care Ultrasound with Functional, Mobility and Frailty Status in Older Inpatients
Source: J Clin Med. 2025 Feb 27;14(5):1603. doi: 10.3390/jcm14051603 (PMC11900600; doi:10.3390/jcm14051603)
Supplement: Supplementary file 1 [file jcm-14-01603-s001.zip › jcm-3416707-supplementary.pdf]

**Supplmentary Information to Article**

# Title: Discriminative ability and associations of sarcopenia using point-of-care ultrasound with functional, mobility and frailty status in older inpatients

**Authors: Rahel Zehnder, Martin Schimmel, Lisa Meyer, Miriam Kömeda, Andreas Limacher, Anna K. Eggimann**

**Date: 02.02.2025**

**Figure S1. Ultrasonographic rectus femoris parameters stratified by sarcopenia status and by sex (n=151)**

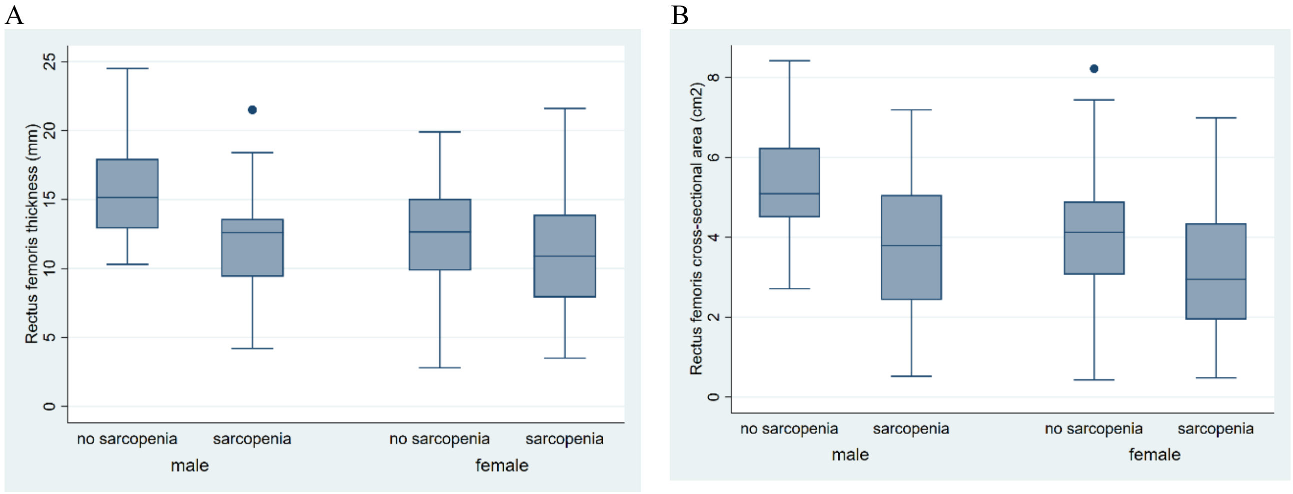


(n=10 missing, BIA could not be performed due to contraindications)
(A) rectus femoris thickness
(B) rectus femoris Cross-sectional area

**Figure S2. Bland Altman Plots of repeatability of measurements of POCUS (intra-rater)**

**Panel A. Rectus femoris thickness**

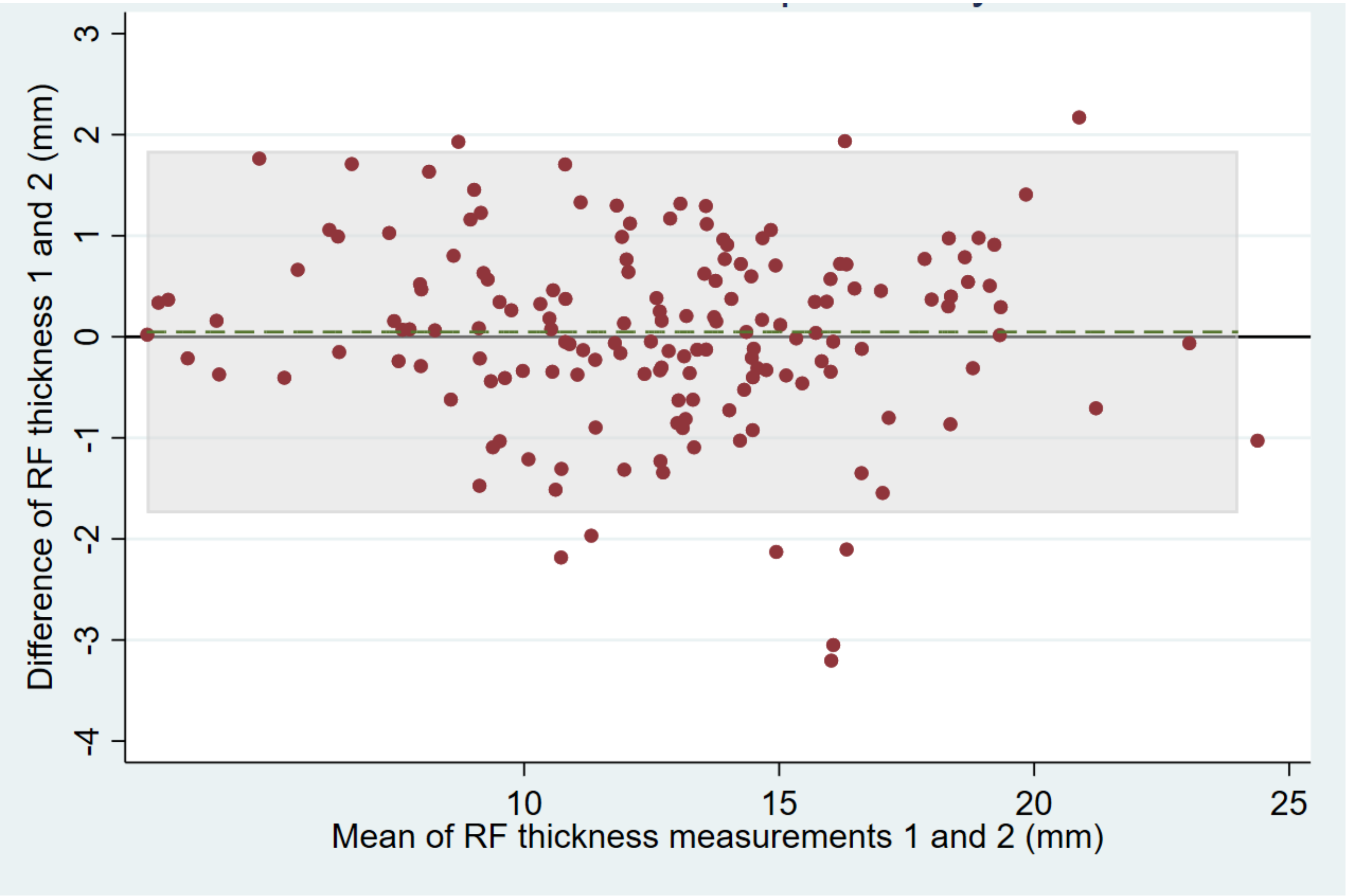

Panel B. Rectus femoris Cross-sectional area (CSA)

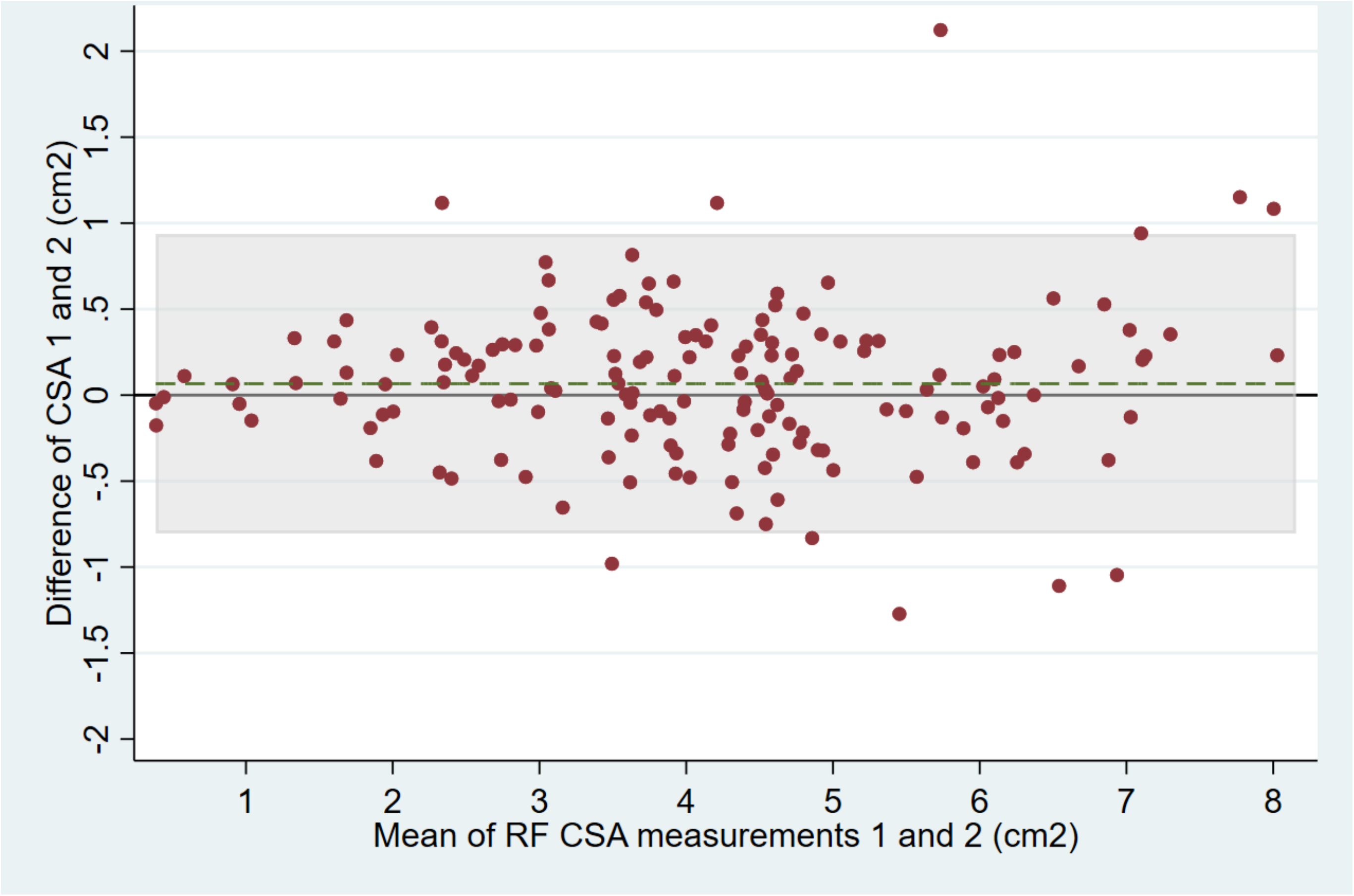

**Table S1. Sensitivity analyses. Association of sarcopenia measures applying Youden cut-offs[e)] with impaired functional status, frailty, and impaired mobility status upon admission (n=161)**

| | Unadjusted OR (95% CI) | Adjusted OR (95% CI) [d)] |
|---|---|---|
| **Sarcopenia based on ultrasound rectus femoris thickness applying Youden cut-off [e)]** | | |
| Impaired functional status [a)] | 6.7 (2.7-16.7) | 6.6 (2.5-17.1) |
| Frailty [b)] | 3.7 (1.5-8.9) | 3.4 (1.4-8.3) |
| Impaired mobility status [c)] | 2.3 (0.9-5.7) | 2.6 (1.0-6.8) |
| **Sarcopenia based on ultrasound rectus femoris cross-sectional area applying Youden cut-off [e)]** | | |
| Impaired functional status [a)] | 9.2 (3.7-22.6) | 9.2 (3.6-23.7) |
| Frailty [b)] | 4.6 (2.0-10.9) | 4.3 (1.8-10.4) |
| Impaired mobility status [c)] | 2.1 (0.9-5.1) | 2.4 (0.9-5.8) |

*Abbreviations: n, number; OR, odds ratio; CI, confidence interval; AUC, area under the curve; RF, rectus femoris; CSA, cross-sectional area*

a) Impaired functional status defined as extended Barthel index ≤31.5 points upon admission. N=5 missing
b) Frailty defined as Clinical Frailty Scale ≥5 points
c) Impaired mobility status defined as DEMMI ≤40 points upon admission. N=14 missing
d) Adjusted for age (continuous) and sex (female vs. male)
e) Youden index derived cut-offs were RF thickness <=13.7 mm for males, and <=11.5mm for females, and RF CSA <=4.4cm 2 for males, and <=3.1cm2 for females. See Methods section
